# Processing of Fluorescent Proteins May Prevent Detection of Prion Particles in [*PSI*^+^] Cells

**DOI:** 10.3390/biology11121688

**Published:** 2022-11-22

**Authors:** Andrew G. Matveenko, Varvara E. Ryzhkova, Natalia A. Zaytseva, Lavrentii G. Danilov, Anastasia S. Mikhailichenko, Yury A. Barbitoff, Galina A. Zhouravleva

**Affiliations:** 1 Department of Genetics and Biotechnology, St. Petersburg State University, 199034 St. Petersburg, Russia; 2 Laboratory of Amyloid Biology, St. Petersburg State University, 199034 St. Petersburg, Russia

**Keywords:** yeast, prion, [*PSI*^+^], Sup35, GFP, RFP, mCherry

## Abstract

**Simple Summary:**

Prions are self-perpetuating protein aggregates that cause fatal neurodegenerative diseases in humans and other mammalian species. In yeast, in contrast to mammals, prions can be maintained in the cell population and confer adaptive traits. Fluorescence microscopy is commonly used to visualize prion aggregates in living cells, providing important information regarding the morphology and localization of prion particles in the cell. In most studies, various constructs with green fluorescent protein (GFP) are used to detect particles of the most-studied yeast prion, [*PSI*^+^]. In our work, we tried to substitute GFP with two different red fluorescent protein variants to expand the application of prion particle imaging. Surprisingly, we found that the processing of the fluorescently labeled prionogenic protein can prevent the detection of prion particles. This pattern was observed for one of the studied red fluorescent proteins (mCherry) and was not dependent on any tested protein degradation systems. The present work thus highlights the limitations of aggregate labeling with fluorescent proteins and suggests labeling with mCherry should be avoided.

**Abstract:**

Yeast is a convenient model for studying protein aggregation as it is known to propagate amyloid prions. [*PSI*^+^] is the prion form of the release factor eRF3 (Sup35). Aggregated Sup35 causes defects in termination of translation, which results in nonsense suppression in strains carrying premature stop codons. N-terminal and middle (M) domains of Sup35 are necessary and sufficient for maintaining [*PSI*^+^] in cells while preserving the prion strain’s properties. For this reason, Sup35NM fused to fluorescent proteins is often used for [*PSI*^+^] detection and investigation. However, we found that in such chimeric constructs, not all fluorescent proteins allow the reliable detection of Sup35 aggregates. Particularly, transient overproduction of Sup35NM-mCherry resulted in a diffuse fluorescent pattern in the [*PSI*^+^] cells, while no loss of prions and no effect on the Sup35NM prion properties could be observed. This effect was reproduced in various unrelated strain backgrounds and prion variants. In contrast, Sup35NM fused to another red fluorescent protein, TagRFP-T, allowed the detection of [*PSI*^+^] aggregates. Analysis of protein lysates showed that Sup35NM-mCherry is actively degraded in the cell. This degradation was not caused by vacuolar proteases and the ubiquitin-proteasomal system implicated in the Sup35 processing. Even though the intensity of this proteolysis was higher than that of Sup35NM-GFP, it was roughly the same as in the case of Sup35NM-TagRFP-T. Thus, it is possible that, in contrast to TagRFP-T, degradation products of Sup35NM-mCherry still preserve their fluorescent properties while losing the ability to decorate pre-existing Sup35 aggregates. This results in diffuse fluorescence despite the presence of the prion aggregates in the cell. Thus, tagging with fluorescent proteins should be used with caution, as such proteolysis may increase the rate of false-negative results when detecting prion-bearing cells.

## 1. Introduction

Baker’s yeast *Saccharomyces cerevisiae* is commonly used as a convenient model organism for studying protein misfolding and aggregation (reviewed in [1]). An important aspect of yeast biology is the existence of yeast prions—self-perpetuating protein aggregates that confer changes to the yeast’s phenotype and are inherited in a non-Mendelian fashion (for review, see [2]). Most yeast prion aggregates have amyloid nature, i.e., their prion conformers form long fibrillar structures with a typical regular structure (for review, see [3,4,5]).

[*PSI*^+^] is the most-studied yeast prion (reviewed in [6]). [*PSI*^+^] is a prion form of the translation termination factor Sup35 [7]. The Sup35 protein contains three major domains. The N-terminal domain (or N-domain) is the prion domain that is required for the formation of an amyloid fibril core and for prion formation [8]. The C-terminal domain (C-domain) is responsible for the termination factor activity of Sup35 [9,10]. Finally, the M-domain, located in the middle of the protein, contributes to the formation of liquid Sup35 droplets [11] and contributes to prion propagation, presumably due to the modulation of prion-chaperone interactions (for review, see [12]). Overproduction of the Sup35 fragment containing the N- and M-domains (Sup35NM) is sufficient for both prion induction and maintenance [13]. The de novo induction of [*PSI*^+^] also depends on the presence of another prion determinant, [*PIN*^+^], the prion form of the Rnq1 protein [14,15].

The presence of [*PSI*^+^] leads to inactivation of the large fraction of the Sup35 protein due to its inclusion into amyloid aggregates. This, in turn, causes a stop codon readthrough that manifests as nonsense suppression, i.e., the ability of the cell to synthesize a full-length protein molecule despite the presence of a premature stop codon in the corresponding gene (for review, see [2]). Different variants (strains) of the [*PSI*^+^] prion vary in the strength of the nonsense suppression, and this variation is explained by the differences in the amount of soluble protein in the cytosol [16]. Excessive aggregation of Sup35, e.g., during its additional production in [*PSI*^+^] cells, can also lead to prion-dependent lethality [14,17].

Apart from the phenotypic analysis, several methods for the detection of [*PSI*^+^] aggregates in the yeast cells have been developed. Most of them (e.g., SDD-AGE [18,19]) are based on the stability and detergent resistance of the prion aggregates. While it is convenient to use such methods to study prions, it is also important to be able to visualize the aggregates directly.

A very suitable technique to observe aggregates in live cells is fluorescence microscopy, as it allows both to quantify the aggregation rate and localize the aggregates in the cellular compartments. For the [*PSI*^+^] detection, Sup35NM fused to the green fluorescent protein (GFP) is often used (reviewed in [20]). The resulting proteins, when transiently produced, are able to be incorporated into pre-existing aggregates [21]. Overproduced Sup35NM-GFP can also form aggregates de novo in the presence of [*PIN*^+^]. Newly formed aggregates may also differ from pre-existing ones by the shape and dynamics of their formation. Particularly, ring- or rod-shaped aggregates are almost exclusively found during Sup35NM overproduction in [*psi*^-^][*PIN*^+^] cells ([22]; see review [2,23]).

Despite the advantages of fluorescent methods, fluorescent proteins other than GFP are rarely used for Sup35 or Sup35NM tagging. However, the development of an efficient construct for the non-GFP labeling of prion particles is crucial for the analysis of the colocalization of prion aggregates with a variety of proteins. At the same time, it is not known whether Sup35 or Sup35NM fused to other fluorescent proteins behave in a manner similar to GFP-containing fusions. In our work, we show that one of the red fluorescent protein variants, mCherry, is not suitable for the visualization of [*PSI*^+^] due to proteolytic processing of the Sup35NM-mCherry protein. At the same time, substitution of GFP for TagRFP-T in a Sup35NM-bearing construct allows for the efficient detection of [*PSI*^+^] particles and has a similar localization pattern compared to commonly used Sup35NM-GFP.

## 2. Materials and Methods

### 2.1. Plasmids

The plasmids pRS316CNMG [24] and pR16CUP-NM-yTagRFP-T [25], in which *SUP35NM* was fused to the *GFP* and *yTagRFP-T* (yeast codon-optimized gene encoding for Tag-RFP-T) genes, respectively, have been described previously. pRS316CG [24] and pRS315CG [25] were used for the *GFP* expression as controls.

pRS315CNMmC and pRS315CmC plasmids for the copper-inducible production of Sup35NM-mCherry and mCherry, respectively, were constructed by replacing GFP sequences with the mCherry gene amplified from a pAG415GPD-Sis1-mCherry vector [26] using the primers EXFP-F-SacII (TGTTACCGCGGATGGTGAGCAAGGGCGAG) and EXFP-R-SacI (GAATCTGAGCTCTTACTTGTACAGCTCGTCCATGCC) and cloning of the SacII and SacI-digested fragment in between the same sites of pRS315CNMG [25] and pRS315CG.

The pR15CUP-NM-yTagRFP-T plasmid was obtained by replacing the PvuI-PvuI fragment, containing a *URA3* selective marker, in pR16CUP-NM-yTagRFP-T with a similar fragment from pRS315 with the *LEU2* marker. The pR15CUP-yTagRFP-T vector was obtained by replacing the BamHI-MroNI fragment in pRS315CG with a similar fragment from pIM35 [27], causing the replacement of the *GFP* sequence by the *yTagRFP-T* gene and the *CYC1* terminator. pIM35 was a kind gift from I. Malcova. The linker between Sup35NM and fluorescent protein in all the Sup35NM-GFP constructs, as well as Sup35NM-mCherry construct, is Pro-Arg. Sup35NM-TagRFP-T constructs have a different 2 aa-long linker, Leu-Asp.

The YCplac33-SIS1 plasmid was constructed by cloning the BamHI-SalI fragment of the pRS315SIS1 [28] plasmid into the YCplac33 vector [29]. Plasmids pRS315, pRS316 [30], pRS315CUP-NM-M0-GFP [25], pRS315CUP-sup35-240-GFP [31], pU-SFP1-GFP [32], and pAG415ADH1-Sis1-EGFP [26] were also used. All the plasmids used in this work are listed in Table S1.

### 2.2. Yeast Strains

Yeast strains used in this work are listed in Table 1.

Most of the experiments were performed using strains isogenic to 74-D694 [33]. The prb1Δ0-P-74-D694 strain was a gift from A. Alexandrov. It maintains a phenotypically weak [*PSI*^+^] variant and contains a complete deletion of *PRB1* constructed as described [37]. The *pep4*Δ strain, yAO121 [38], was a gift from Y.O. Chernoff’s lab. The strain P2.1.1-yAO121 was obtained by Sup35NM-GFP overproduction and subsequent selection of a strong suppressor strain using the color phenotype on 1/4YEPD media. The [*psi*^-^][*pin*^-^] strains 2-74-D694, prb1Δ0-2-74-D694, and 2-yAO121 were obtained after 3 passages on the GuHCl-containing medium of their respective ancestor strains, 74-D694, prb1Δ0-P-74-D694, and yAO121.

P1-U-1A-D1628 was obtained as a spontaneously appearing white colony in U-1A-D1628 [42,43] progeny. 3.1P.1-1B-D1606 was selected as a [*PSI*^+^]-containing clone after the co-expression of *SUP35NM* and *RNQ1* in 1B-D1606 [42]. Ψ1-33G-D373 [39] is a Ψ-33G-D373 subclone which demonstrated higher levels of nonsense-suppression. U-T-P^T^-YAL2171 is a T-P^T^-YAL2171 [12] derivative, in which *SIS1*-containing plasmid was substituted for the YCplac33-Sis1 via plasmid shuffling. The presence of the [*PSI*^+^] prion in all strains was confirmed by loss of the suppressor phenotype after passaging on the GuHCl-containing media, as well as by the appearance of fluorescent foci after transient overexpression of Sup35NM-GFP.

### 2.3. Media and Cultivation

Standard media and methods for yeast cultivation were used [44,45]. 1/4YEPD medium [46] was used for color selection. To obtain strains without prions, yeast colonies were passed three times on either YEPD of 1/4YEPD plates containing 4mM GuHCl. Yeast transformations were performed using a standard protocol [47]. For the induction of the *CUP1* promoter, copper sulfate was added to the media at a final concentration of 50 μM. The experiments involving the inhibition of proteasomes were performed as described previously [48]. DMSO-diluted MG132 (Sigma, #C2211-5MG) was used at a final concentration of 40 μM.

### 2.4. Fluorescence Microscopy

Fluorescence was visualized in living cells cultivated as described previously [32]. Wide-field fluorescence microscopy was performed using a Zeiss Axioscope A1. Filter sets 63HE, 38, and 74HE were used to visualize green, red, and green+red fluorescence, respectively. A Leica TCS SP5 MP was used for confocal microscopy. Confocal images were analyzed using ImageJ [49]. Cell counts for various strains producing Sup35NM-GFP and Sup35NM-mCherry were compared using Fisher’s exact test. Data analysis and visualization was performed in R [50].

### 2.5. Protein Analysis

To isolate total protein from yeast cells, we used a standard alkaline lysis protocol [51]. To isolate proteins from yeast cells with preserved aggregates, we used a glass bead lysis technique [52] with a lysis buffer (100 mM Tris-HCl (pH 7.5), 50 mM NaCl, 2 mM PMSF, and 10 mM BME) [18]. SDS-PAGE, semi-dry transfer and Western blotting were performed using standard protocols [44]. For experiments related to the visualization of proteins in a gel (SDS-PAGE), we used a modified protocol with 10% separating and 4% concentrating polyacrylamide gels.

Anti-Sup35NM primary antibodies SE4291 [53] were used. Anti-rabbit antibodies from the ECL Plus Western Blotting Detection System kit (Amersham) were used as secondary antibodies. The signal was detected using the ECL Plus Western Blotting Reagent Pack (Amersham) and GeneGnome (SynGene) hardware and software.

To visualize fluorescently tagged proteins directly in the gel, we used a GE Typhoon FLA 9500 instrument with Typhoon scanner software (GE Healthcare (Buckinghamshire, UK)). To visualize GFP-labeled proteins, we used the Alexa Fluor 488 filter set (473 nm excitation, ≥510 nm emission). To visualize TagRFP-T or mCherry-labeled proteins, we used a Cy3 filter set (532 nm excitation, 560–580 nm emission). We also used a Cy5 filter set (635 excitation, ≥665 nm emission) to visualize a molecular weight marker and for the auto-fluorescence control.

## 3. Results

### 3.1. Sup35NM-mCherry Does Not Allow Detection of the *[PSI*^+^*]* Aggregates

**Figure 1:**
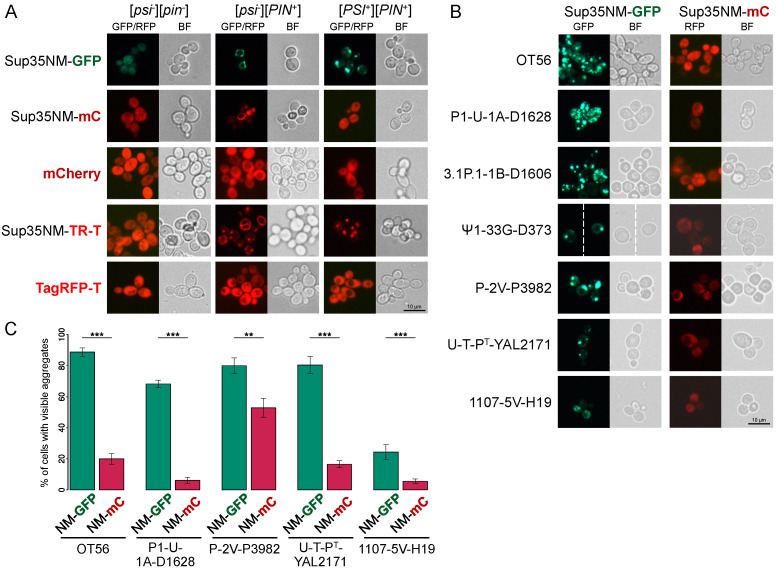
Fusion of Sup35NM to mCherry does not allow the detection of [*PSI*^+^] aggregates. Clones expressing copper-inducible constructs in OT56 ([*PSI*^+^][*PIN*^+^]), 1-OT56 ([*psi*^-^][*PIN*^+^]), 2-OT56 ([*psi*^-^][*pin*^-^]) (**A**), and other [*PSI*^+^] strains (**B**) were analyzed with fluorescence microscopy. Shown are representative images of either green (GFP) or red (RFP) fluorescence acquired using respective filter sets. Scale bar equals 10 μM. Cells producing Sup35NM-mCherry and Sup35NM-TagRFP-T are henceforth denoted as Sup35NM-mC and Sup35NM-TR-T, respectively. (**C**). Quantification of the frequencies of the fluorescent foci detection in cells from B (see Supplementary Table S2 for the detailed cell count data). NM stands for Sup35NM. **, *p* < 0.01; ***, *p* < 0.001 in Fisher’s exact test. Error bars indicate percentage error.

In order to visualize [*PSI*^+^] aggregates tagged with a red fluorescent protein, we substituted the GFP-encoding sequence for the mCherry and thus obtained a plasmid for transient Sup35NM-mCherry production. To our surprise, Sup35NM-mCherry demonstrated diffuse fluorescence in the [*PSI*^+^] cells (Figure 1A) despite the same media, growth conditions, and the identical induction time for the Sup35NM-GFP and Sup35NM-mCherry. In all cases, diffuse fluorescence was observed in the [*psi*^-^][*pin*^-^] strains; however, we did observe ring-shaped Sup35NM-mCherry aggregates in the [*psi*^-^][*PIN*^+^] cells (Figure 1A). The appearance of similar ring- and dot-shaped aggregates of Sup35NM-GFP is a known hallmark of de novo [*PSI*^+^] induction in [*PIN*^+^] strains [22].

Initially, we tested two isogenic [*PSI*^+^] strains, OT56 and P-74-D694, bearing different prion variants, with identical results. Since both strains are 74-D694 derivatives, to find out whether this effect is inherent to the 74-D694 background, we tested various [*PSI*^+^] derivatives, which were obtained independently in laboratory strains of different origins. In all cases, we found diffuse Sup35NM-mCherry, while Sup35NM-GFP formed aggregates in the same strains under the same conditions (Figure 1B). Quantification confirmed that in all strains, cells with detectable aggregates were significantly less frequent in the case of Sup35NM-mCherry compared to Sup35NM-GFP (Figure 1C, Supplementary Table S2). Of all the strains, only P-2V-P3982 demonstrated a comparatively high rate of Sup35NM-mCherry aggregate appearance, which was still lower than that of Sup35NM-GFP. The pattern of fluorescence was also different, as most of the cells producing Sup35NM-mCherry displayed multiple small foci, while Sup35NM-GFP more often formed one or several large aggregates (Supplementary Table S2).

As an alternative to mCherry, we next used another red fluorescent protein, TagRFP-T. Sup35NM-TagRFP-T demonstrated a fluorescence pattern similar to Sup35NM-GFP and formed visible aggregates in the [*PSI*^+^] strains (Figure 1A). Aggregates visualized using Sup35NM-TagRFP-T demonstrated similar properties to Sup35NM-GFP. Particularly, they partially colocalized with GFP-labeled aggregates of QN-rich transcription factor Sfp1 and with an Hsp40 chaperone Sis1 (Supplementary Figure S1), as was previously shown for the Sup35NM-GFP [32]. Moreover, using the Sup35NM-TagRFP-T construct, we showed that wild-type Sup35NM colocalizes with Sup35NM-M0 and Sup35-240 variants (Supplementary Figure S2). These mutations are good model systems for assessing the effects of aggregation since they have been shown to destabilize [*PSI*^+^] by co-aggregating with native Sup35 [25,31]. Evidently, Sup35NM-TagRFP-T and Sup35NM-GFP did also colocalize with each other (Supplementary Figure S2). None of the aforementioned interactions could have been clearly shown using Sup35NM-mCherry. It is thus evident that TagRFP-T and GFP are suitable fluorescent markers for Sup35NM, while mCherry is not.

### 3.2. Fusion to mCherry Does Not Affect Sup35NM Prion Properties

**Figure 2:**
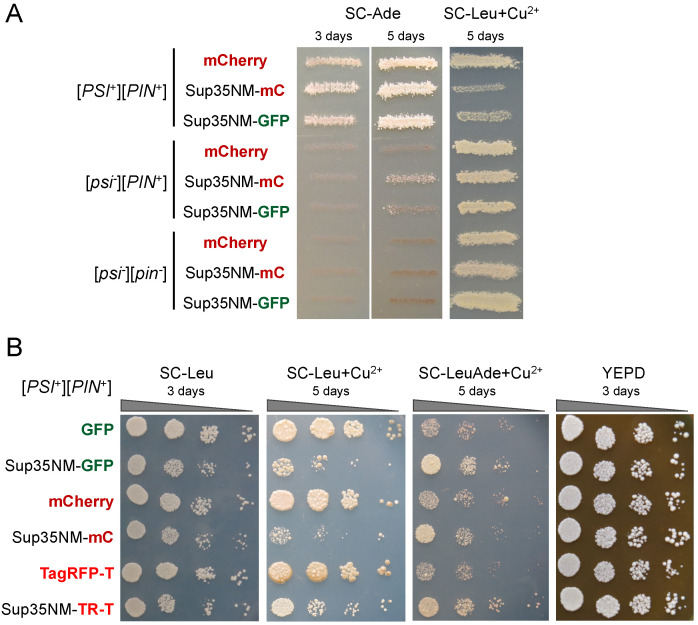
Sup35NM-mCherry protein retains the prion properties and does not cause the loss of [*PSI*^+^]. (**A**). Phenotypic assay performed by replica plating of OT56 [*PSI*^+^][*PIN*^+^], 1-OT56 ([*psi*^-^][*PIN*^+^]), and 2-OT56 ([*psi*^-^][*pin*^-^]) strains, transformed with vectors pRS315CmC, pRS315CNMmC, and pRS315CNMG for copper-inducible expression of *mCherry*, *SUP35NM-mCherry*, and *SUP35NM-GFP*, respectively. Similar to Sup35NM-GFP, Sup35NM-mCherry enhances nonsense-suppression and prion toxicity in the [*PSI*^+^][*PIN*^+^] strain and promotes de novo [*PSI*^+^] formation in the [*psi*^-^][*PIN*^+^] strain. (**B**). Tenfold serial dilutions of the OT56 transformed with vectors from A, as well as pRS315CG, pR15CUP-NM-yTagRFP-T, and pR15CUP-yTagRFP-T for the inducible expression of *GFP*, *SUP35NM-yTagRFP-T*, and *yTagRFP-T*, respectively.

The most obvious explanation for the unusual behavior of Sup35NM-mCherry would be mCherry affecting Sup35NM properties leading to either the loss of [*PSI*^+^] or the inability of Sup35NM to coaggregate with [*PSI*^+^] prion particles. We tested the effects of transient overproduction of Sup35NM-mCherry on [*PSI*^+^]’s properties. We assessed the enhancement of the nonsense-suppressor phenotype and prion toxicity in [*PSI*^+^] strains as well as the ability to induce [*PSI*^+^] in [*psi*^-^][*PIN*^+^] strains. In all cases, Sup35NM-mCherry behaved similarly to Sup35NM-GFP (Figure 2A), indicating that mCherry did not significantly alter Sup35NM prion properties. Similar results were obtained for the Sup35NM-TagRFP-T, as it enhanced suppression and prion toxicity in [*PSI*^+^][*PIN*^+^] strains (Figure 2B) and promoted de novo [*PSI*^+^] formation in [*psi*^-^][*PIN*^+^] strains.

### 3.3. Sup35NM-mCherry and Sup35NM-TagRFP-T Are Partially Degraded in Yeast Cells

**Figure 3:**
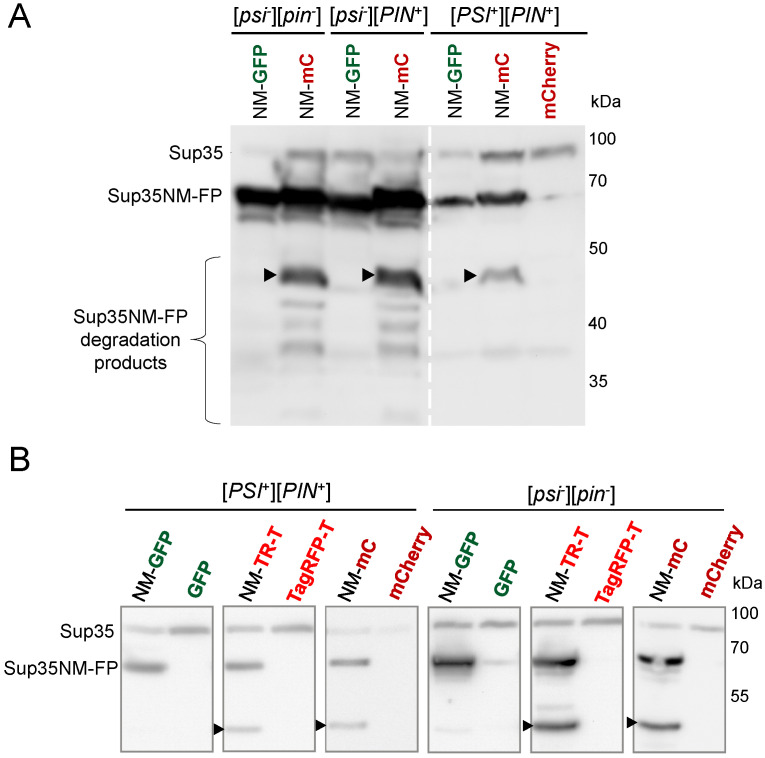
Both Sup35NM-TagRFP-T and Sup35NM-mCherry, but not Sup35NM-GFP, are subjected to proteolysis in yeast cells. (**A**,**B**) Western blot analysis of protein lysates from clones shown in Figure 2A and Figure 1A, respectively. Arrowheads indicate major additional product detected by anti-Sup35NM antibodies, which is similar in weight to the untagged Sup35NM. FP—fluorescent protein (GFP, or mCherry, or TagRFP-T).

We analyzed protein extracts from [*PSI*^+^] and [*psi*^-^] cells expressing various Sup35NM-FP constructs with Western blotting. Sup35NM-mCherry demonstrated multiple bands visualized using anti-Sup35NM antibodies (Figure 3A). The most abundant additional product appeared similar in weight to the untagged Sup35NM. All these degradation products were not observed in the case of Sup35NM-GFP.

We thus hypothesized that such enhanced degradation of Sup35NM-mCherry was the reason it was unable to decorate [*PSI*^+^] aggregates. However, the same analysis revealed that Sup35NM-TagRFP-T, which showed a normal aggregation pattern, also degraded abundantly when overproduced under the same conditions as other constructs, which contradicts this hypothesis (Figure 3B). Consequently, Sup35NM-mCherry degradation is not the primary cause of its inability to visualize [*PSI*^+^] aggregates.

### 3.4. Products of Sup35NM-mCherry Degradation Retain Fluorescent Properties thus Interfering with Detection of the [*PSI*^+^] Aggregates

**Figure 4:**
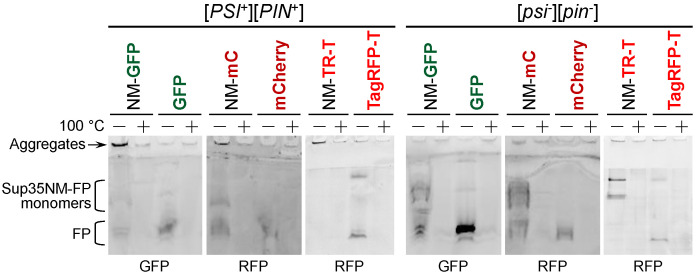
Sup35NM-mCherry degradation leads to decrease in relative amount of aggregates detectable by fluorescence. Proteins from OT56 ([*PSI*^+^] [*PIN*^+^]) or 2-OT56 ([*psi*^-^] [*pin*^-^]) cells expressing indicated constructs were extracted in non-denaturing conditions. After addition of the SDS-containing buffer, samples were either boiled for 5 min (+) or incubated at room temperature (−). SDS-PAGE was visualized using a gel documentation system with filter sets for either green (GFP) or red (RFP) fluorescence.

As visible fluorescence of mCherry can be observed during transient overproduction of the Sup35NM-mCherry, it is possible that at least some of the Sup35NM-mCherry degradation products retain fluorescent properties. We next attempted to determine whether fluorescent products of Sup35NM-mCherry exist in aggregated or monomeric form. After protein extraction in non-denaturing conditions, we performed SDS-PAGE and visualized fluorescence of the whole gel. We first confirmed that SDS treatment resulted in a loss of fluorescence of neither GFP nor mCherry, while the same samples exhibited no fluorescence after heating to 100 ^°^C (Figure 4). We were also able to distinguish between aggregated and monomeric Sup35NM-FP as large aggregates are stuck in wells in the upper gel. Consistent with Sup35 being mostly in aggregated form in [*PSI*^+^] [*PIN*^+^] cells, we observed a smaller amount of monomeric Sup35NM-FP as compared to [*psi*^-^] [*pin*^-^] cells. At the same time, Sup35NM-FP aggregates were visible in [*PSI*^+^] [*PIN*^+^] cells; however, the fluorescing aggregates were much more abundant in the case of Sup35NM-GFP, indicating that Sup35NM-mCherry degradation greatly reduces the amount of the full-length protein that remains in an aggregated state. At the same time, fluorescing products of Sup35NM-mCherry processing were also more abundant than analogous products of Sup35NM-GFP (Figure 4). In contrast, Sup35NM-TagRFP-T showed a distribution of fluorescent aggregates and monomers similar to Sup35NM-GFP (Figure 4), indicating that, despite the similar degradation pattern of Sup35NM-mCherry and Sup35NM-TagRFP-T, the resulting products of the latter do not retain fluorescent properties. These observations suggest that enhanced Sup35NM-mCherry degradation may lead to a reduction in aggregate fluorescence and an increase in the fluorescence of monomeric proteins with presumably diffuse distribution in the cytosol, which can explain the unreliability of using Sup35NM-mCherry for [*PSI*^+^] detection.

### 3.5. Degradation of Sup35NM-mCherry and Sup35NM-TagRFP-T Is Not Caused by Vacuolar Proteases

**Figure 5:**
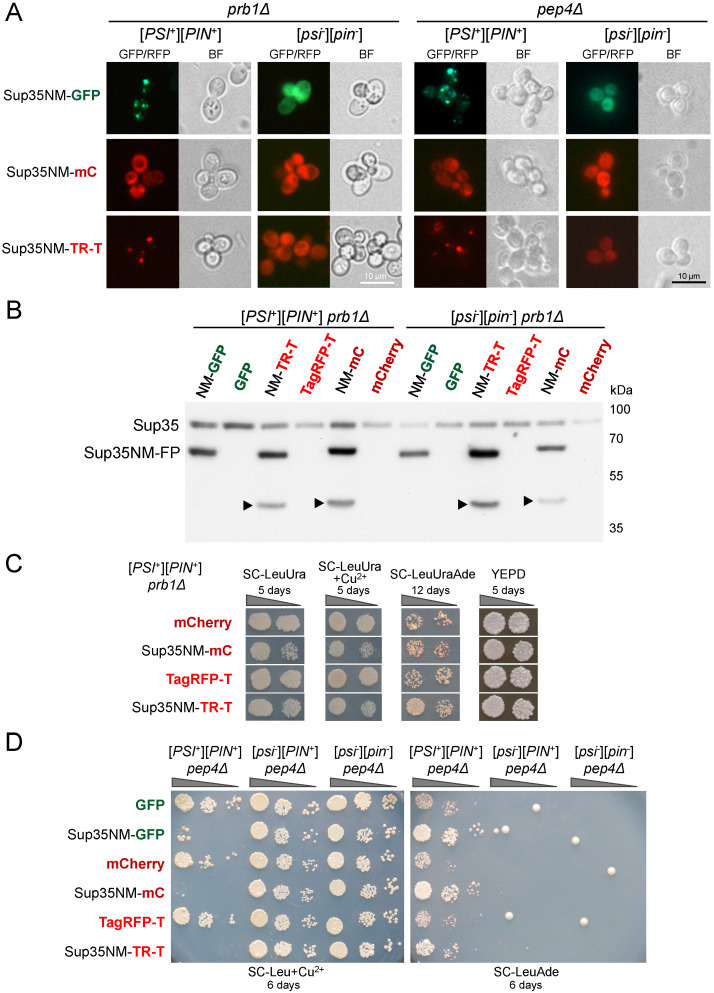
Vacuolar proteases do not influence Sup35NM-mCherry properties. (**A**). Fluorescent images of the *prb1*Δ or *pep4*Δ cells overexpressing *SUP35NM* fused to different fluorescent protein genes. (**B**). Western blot analysis of protein lysates from *prb1*Δ strains overproducing the indicated proteins. Arrowheads indicate major additional products detected by anti-Sup35NM antibodies. (**C**). Tenfold serial dilutions of the prb1Δ0-P-74-D694 strain co-transformed either with pRS315 together with pIM35 (TagRFP-T) or pR16CUP-NM-yTagRFP-T (Sup35NM-TR-T), or with pRS316 together with pRS315CmC (mCherry) or pRS315CNMmC (Sup35NM-mC). The panels for each medium are taken from the same plate. (**D**). Tenfold serial dilutions of the *pep4*Δ strains transformed with plasmids for expression of the indicated constructs.

As vacuolar protease B is known to affect Sup35 stability [38], we tested whether the presence of Prb1 affects the ability of Sup35NM-mCherry to visualize preexisting [*PSI*^+^] aggregates. We used strains isogenic to 74-D694 with deleted *PRB1* and found no influence on the fluorescence pattern of Sup35NM-mCherry (Figure 5A). We also found that Prb1 did not cause the increased degradation of Sup35NM-mCherry (Figure 5B) and did not affect its ability to enhance [*PSI*^+^] prion phenotype (Figure 5C). Similar results were obtained for the vacuolar protease A, encoded by the *PEP4* gene: its deletion did not affect Sup35NM-mCherry’s fluorescence pattern (Figure 5A) or the prion properties of Sup35NM (Figure 5D). Although we observed some variation between individual transformants in strength of the Sup35NM-FP effects on phenotype, we consistently observed enhanced suppression and prion toxicity in all cases when Sup35NM-FP constructs were produced in [*PSI*^+^] strains.

### 3.6. Sup35NM-mCherry Degradation Is Not Affected by the Ubiquitin-Proteasomal Pathway

**Figure 6:**
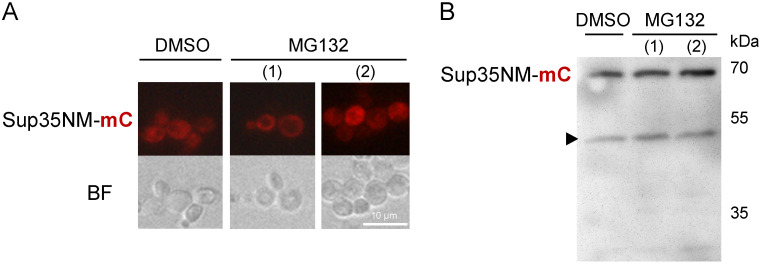
Proteasomal degradation is not responsible for the Sup35NM-mCherry processing and diffuse distribution in [*PSI*^+^] cells. OT56 cells expressing *SUP35NM-mCherry* under control of *CUP1* promoter were treated with MG132 for 4 hours; DMSO treatment was used as a control. MG132 was either added simultaneously with CuSO_4_ (1) or one hour prior to CuSO_4_ addition (2). Cells were analyzed with fluorescence microscopy (**A**) or were subjected to protein extraction and Western blotting (**B**). Anti-Sup35NM antibodies were used.

Many misfolded proteins are known substrates of the ubiquitin-proteasomal degradation pathway. To find out whether Sup35NM-mCherry is degraded by the cytoplasmic proteasome, we used the proteasomal inhibitor MG132. After growing cells with Sup35NM-mCherry overproduction in the presence or absence of MG132, we analyzed both fluorescence and protein lysates. However, no influence of the proteasomal inhibition on either diffuse Sup35NM-mCherry distribution (Figure 6A) or its degradation (Figure 6B) could be observed.

## 4. Discussion

The visualization of prion particles in cells is an important technique that is widely used to study different aspects of prion biology. Despite the relative simplicity of the method, the outcome of experiments involving fluorescently labeled prion proteins is known to be possibly dependent on the particular construct. Two key questions usually arise regarding the application of fusion proteins for the detection and characterization of aggregates: (i) does the fusion protein co-aggregate with the native protein of interest? and (ii) is the localization of fusion protein foci similar to the localization of the native prion particles? In this work, we have characterized another important aspect of the problem—proteolytic degradation of the Sup35NM-mCherry fusion protein, which compromises its usage for tracking the prion status of the cell.

In most cases, a GFP tag is used to detect Sup35 prion particles in yeast cells. Two different types of GFP-fused constructs are commonly used for the detection of [*PSI*^+^] particles. The first type contains GFP at the C-terminus of either the full-length or truncated part of the Sup35 protein [24,54]. The second type of construct contains GFP inserted between the N- and M-domains of Sup35 and is thus called NGMC, but it is often referred to as Sup35-GFP [55,56]. Such a construct can efficiently replace the endogenous copy of the *SUP35* gene in the yeast genome, thus avoiding spurious effects imposed by the additional production of Sup35. However, the intracellular localization of the NGMC protein differs from that of the Sup35NM-GFP: while a single fluorescent focus or several large foci are observed upon the overproduction of Sup35-GFP (Sup35NMC-GFP) or Sup35NM-GFP [24], multiple small fluorescent dots are usually seen for NGMC [56]. It has been shown that the single fluorescent focus of Sup35NM-GFP localizes in the peripheral deposit of aggregated proteins, IPOD [57,58]. In our study, we observed a similar localization of both Sup35NM-GFP and Sup35NM-TagRFP-T (Figure 1, as seen in Figure 5). It is thus evident that transient overproduction may lead to the redistribution of the prion particles within the cell.

The usage of anything other than GFP fluorescent proteins for Sup35NM visualization is quite rare. Several studies made use of Sup35NM-DsRed for the co-localization of Sup35 aggregates with other proteins [59,60,61]. However the DsRed protein would be a rather questionable choice as it has a tetrameric structure and has a relatively long maturation time [62]. Despite these limitations, we found only one occasion of using another red fluorescent protein for Sup35NM detection, TagRFP [63], which is an ancestor to the TagRFP-T [62] that we use in the present study. Sup35NM-TagRFP demonstrated a similar fluorescence pattern to Sup35NM-GFP, which corroborates our findings; however, the rationale behind the usage of this specific red fluorescent protein was not presented [63]. Another interesting observation was made by Greene et al. [64], who showed that the substitution of the GFP with RFP (of unknown type) in the NGMC construct dramatically changes the sensitivity of the prion to changes in chaperone levels. Such changes might be caused by increased degradation, similar to the one we observed in this work; however, the authors provided no explanation for their observations.

Overall, the usage of fluorescent proteins other than GFP may be important to enable colocalization assays involving libraries of GFP-labeled proteins. The aforementioned issues with the N-RFP-MC construct, as well as proteolytic processing of the Sup35NM-mCherry observed in our study, makes the task of selecting another fluorescent protein more difficult. Importantly, processing of the fusion protein may occur despite the fact that the expression of the fusion gene displays all the expected phenotypic effects (Figure 2). Notably, the fusion of mCherry to Sup35 fragments has been previously applied [65]. Our data regarding the processing of mCherry fusions questions the validity of conclusions made using mCherry-tagged Sup35 variants. We also demonstrate that the Sup35NM-TagRFP-T construct enables the analysis of prion particles with the same efficiency as the conventional Sup35NM-GFP (Figure 1, as seen Figure 3). Thus, Sup35NM-TagRFP-T is, in our view, the best option to be used for co-localization assays together with non-prion GFP-labeled proteins.

Another important aspect of the fluorescent protein processing problem is the identification of the proteolysis pathway leading to the observed fluorescence pattern. We show that the deletion of neither *PRB1* nor *PEP4* genes compensates for the observed proteolysis of Sup35NM-mCherry (Figure 5). This observation is important in light of previous analyses, which showed that limited proteolysis of Sup35 may proceed through vacuolar proteases Pep4 and Prb1 [38]. Our data suggest that proteolytic processing of the chimeric Sup35NM-mCherry protein may go along some alternative pathway that does not involve vacuolar proteases or cytosolic proteasomes (Figure 6). It is possible that the process involves an unknown protease. An alternative hypothesis suggests that autophagy may be responsible for the degradation of the chimeric protein, for example, due its aberrant aggregation. It has been reported that due to autophagy, pathway cleavage of GFP from fusion proteins, such as EGFP-Atg8 and EGFP-Ede1, may occur [66,67]. Autophagy has also been shown to reduce [*PSI*^+^] formation [68] and affect [*PSI*^+^] curing under certain conditions [69]. It can be hypothesized that Sup35NM-mCherry is cleaved more efficiently than Sup35NM-GFP in [*PSI*^+^] cells. In favor of this hypothesis is our observation that visible aggregates of Sup35NM-mCherry can be detected in [*psi*^-^] [*PIN*^+^] cells during de novo [*PSI*^+^] formation (Figure 1A). It is possible that cells need some time to adapt to the presence of [*PSI*^+^] by enhancing the autophagy or other proteolytic systems capable of degrading Sup35NM-FP. It should be mentioned that we consistently observed smaller amounts of Sup35NM-FP proteins in the [*PSI*^+^] cells as compared to [*psi*^-^] cells, despite identical conditions for their transient overexpression (Figure 3A). Indeed, the presence of additional Sup35NM is more toxic to the [*PSI*^+^] than [*psi*^-^] cells [14,70], so the Sup35NM degradation that we observed may be a manifestation of some mechanism for reducing this toxicity.

## 5. Conclusions

Taken together, our results emphasize the importance of the rational selection of the fluorescent proteins for detection of prion particles and highlight that the Sup35NM-mCherry fusion (and, possibly, other fusion proteins) is not suitable for the analysis of prion particles using fluorescence microscopy.

## Figures and Tables

**Table 1:** Strains of *S. cerevisiae* used in this study.

Strain	Genotype	Background	Reference
74-D694	*MATa ade1-14 his3-*Δ*200 ura3-52 leu2-3,112 trp1-289*[*psi^-^*] [*PIN^+^*]	74-D694	[33]
P-74-D694	*MATa ade1-14 his3-*Δ*200 ura3-52 leu2-3,112 trp1-289*[*PSI^+^*] [*PIN^+^*]	74-D694	[34]
2-74-D694	*MATa ade1-14 his3-*Δ*200 ura3-52 leu2-3,112 trp1-289*[*psi^-^*] [*pin^-^*]	74-D694	This work
OT56	*MATa ade1-14 his3-*Δ*200 ura3-52 leu2-3,112 trp1-289*[*PSI^+^*]*^S^*[*PIN^+^*]	74-D694	[35,36]
1-OT56	*MATa ade1-14 his3-*Δ*200 ura3-52 leu2-3,112 trp1-289*[*psi^-^*] [*PIN^+^*]	74-D694	[32]
2-OT56	*MATa ade1-14 his3-*Δ*200 ura3-52 leu2-3,112 trp1-289*[*psi^-^*] [*pin^-^*]	74-D694	[32]
prb1Δ0-P-74-D694	*MATa ade1-14 his3-*Δ*200 ura3-52 leu2-3,112 trp1-289 prb1*Δ*0*[*PSI^+^*] [*PIN^+^*]	74-D694	[37]
prb1Δ0-2-74-D694	*MATa ade1-14 his3-*Δ*200 ura3-52 leu2-3,112 trp1-289 prb1*Δ*0*[*psi^-^*] [*pin^-^*]	74-D694	This work
yAO121	*MATa ade1-14 his3-*Δ*200 ura3-52 leu2-3,112 trp1-289 pep4::HIS3MX*[*psi^-^*] [*PIN^+^*]	74-D694	[38]
P2.1.1-yAO121	*MATa ade1-14 his3-*Δ*200 ura3-52 leu2-3,112 trp1-289 pep4::HIS3MX*[*PSI^+^*]*^2.1.1^*[*PIN^+^*]	74-D694	This work
2-yAO121	*MATa ade1-14 his3-*Δ*200 ura3-52 leu2-3,112 trp1-289 pep4::HIS3MX*[*psi^-^*] [*pin^-^*]	74-D694	This work
P1-U-1A-D1628	*MATα ade1-14 his3-*Δ*200 lys2 ura3-52 leu2-3,112 trp1-289 sup45::HIS3MX*[pRS316-SUP45] [*PSI^+^*]	1A-D1628	This work
3.1P.1-1B-D1606	*MATα ade1-14 his7-1 lys9-A21 ura3-52 leu2–3,112 trp1-289 gal10-1B*[*PSI^+^*]*^3.1^*	1B-D1606	This work
Ψ1-33G-D373	*MATα ade2-144,717 pha2P-A10 (pheA10) his7-1 lys9-A21 ura3-52 leu2–3,112 trp1-289*[*PSI^+^*]	33G-D373	[39]; this work
P-2V-P3982	*MATα ade1-14 his7-1 lys2-87 ura3*Δ *leu2-B2 thr4-B15*[*PSI^+^*]	2V-P3982	[40]
U-T-P^T^-YAL2171	*MATa ade1-14 his3-11,-15 ura3-1 leu2-3,112 trp1-1 can1-100 sup35::hphMX sis1::kanMX*[pRS314-SUP35] [YCplac33-Sis1] [*PSI^+^*] [*PIN^+^*]	W303	This work
1107-5V-H19	*MATa ade2-1 ura3-52 leu2-3,112 can1-100 SUQ5*[*PSI^+^*] [*PIN^+^*]	S288C	[41]

## Data Availability

All data obtained during this work, as well as vector sequences and R code used for data analysis, are available from the authors upon request.

## Supplementary Materials

The following supporting information can be downloaded at: https://www.mdpi.com/article/10.3390/biology11121688/s1, Table S1. List of plasmids used in this work. Table S2. Quantification of the Sup35NM-GFP and Sup35NM-mCherry fluorescence in various [*PSI*^+^] strains; Figure S1: [*PSI*^+^] aggregates visualized using Sup35NM-TagRFP-T transient overproduction colocalized with Sfp1 and Sis1; Figure S2: Sup35NM-TagRFP-T aggregates colocalize with Sup35NM-M0 and Sup35-240 in [*PSI*^+^] cells. Figure S3: Original Images of Figure 3A; Figure S4: Original Images of Figure 3B(1); Figure S5: Original Images of Figure 3B(2); Figure S6: Original Images of Figure 4-[Alexa Fluor 488]; Figure S7: Original Images of Figure 4-[Cy3]; Figure S8: Original Images of Figure 5B; Figure S9: Original Images of Figure 6B.

## Sample Availability

Strains and plasmids used for experiments are available from the authors.

## Abbreviations

The following abbreviations are used in this manuscript:

aa	Amino acid
GFP	Green fluorescent protein
GuHCl	Guanidine hydrochloride
RFP	Red fluorescent protein
