# Supplementary material for: Processing of Fluorescent Proteins May Prevent Detection of Prion Particles in [PSI+] Cells
Source: Biology (Basel). 2022 Nov 22;11(12):1688. doi: 10.3390/biology11121688 (PMC9774836; doi:10.3390/biology11121688)
Supplement: Supplementary file 1 [file biology-11-01688-s001.zip › Supplementary_Figs S1,S2.pdf]

Processing of fluorescent proteins may prevent detection of prion particles in [*PSI*$^+$] cells

Andrew G. Matveenko, Varvara E. Ryzhkova, Natalia A. Zaytseva, Lavrentii G. Danilov, Anastasia S. Mikhailichenko, Yury A. Barbitoff, Galina A. Zhouravleva

Supplementary Figures

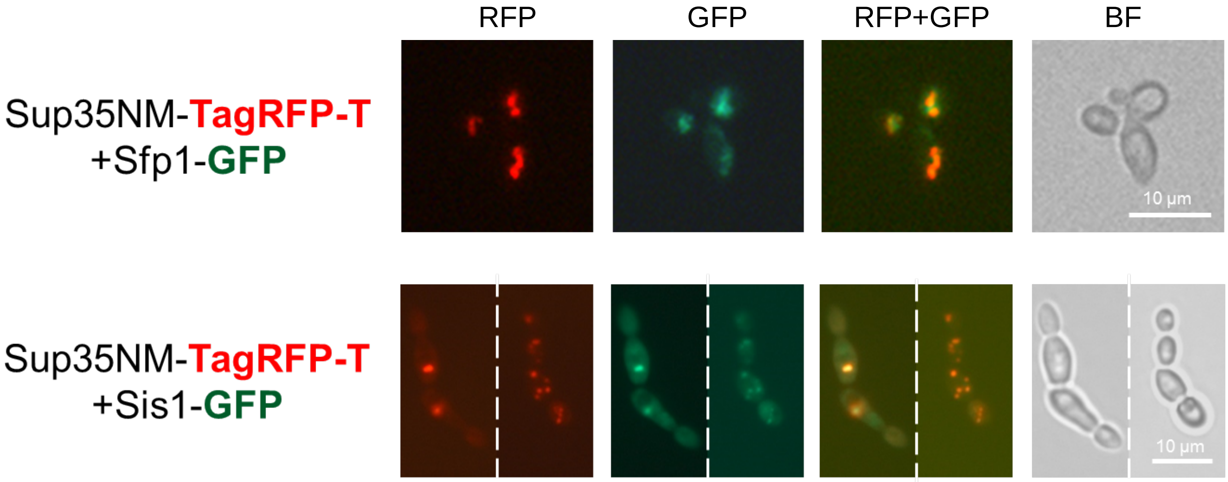

Figure S1. [*PSI*$^+$] aggregates visualized using Sup35NM-TagRFP-T transient overproduction partially colocalize with Sfp1 and Sis1. Shown are representative images of cells of OT56 transformed with pR15CUP-NM-yTagRFP-T and pU-SFP1-GFP plasmids (upper row) and P-74-D694 transformed with pR16CUP-NM-yTagRFP-T and pAG415ADH1-Sis1-EGFP (lower row) after 4 hours of copper induction. Scale bar equals 10 µm.

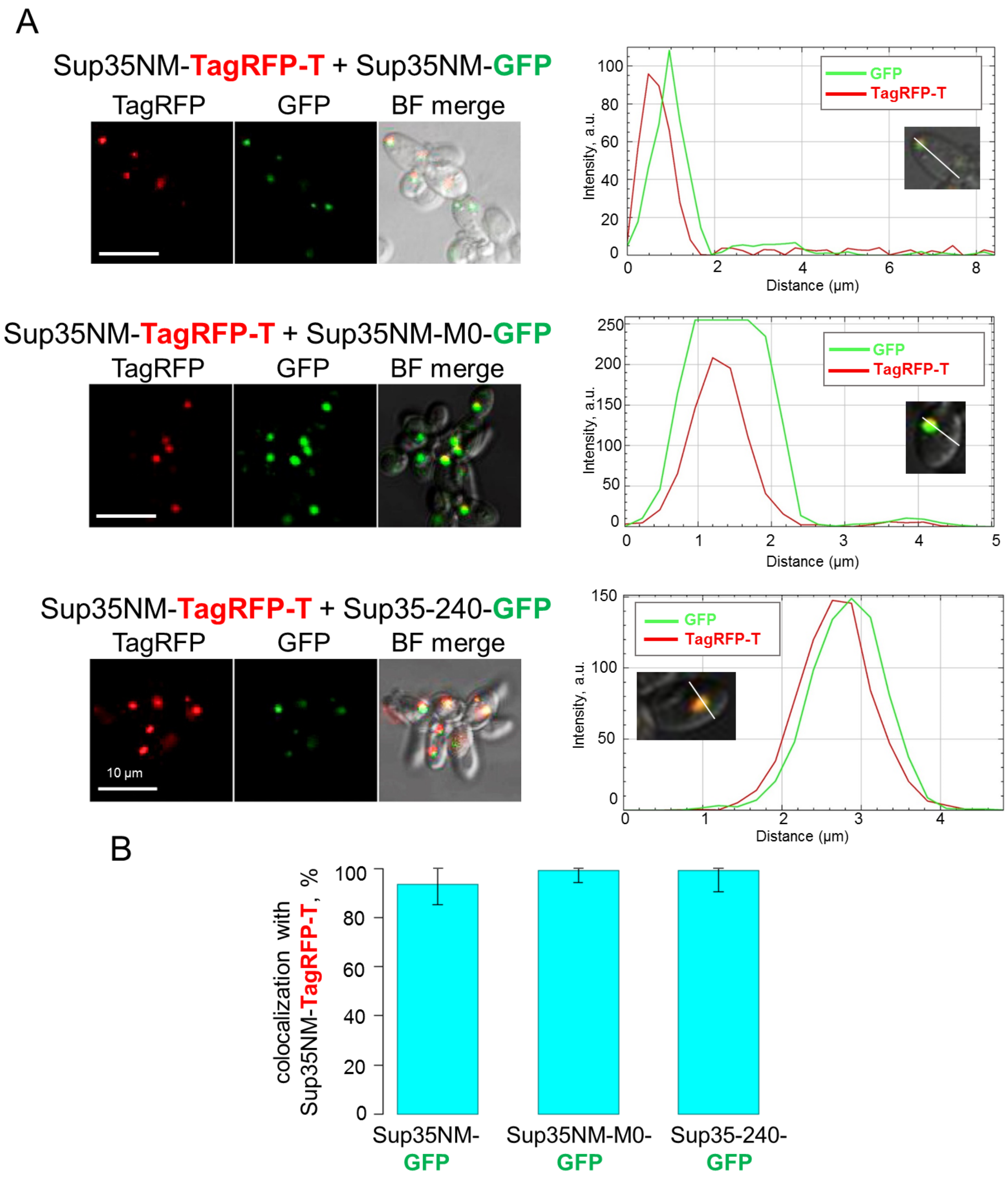

Figure S2. Sup35NM-TagRFP-T aggregates colocalize with Sup35NM-M0 and Sup35-240 in [*PSI*$^+$] cells. A. Confocal images of the representative fields of P-74-D694 cells co-overproducing indicated constructs from plasmids pR16CUP-NM-yTagRFP-T (Sup35NM-TagRFP), pRS315CNMG (Sup35NM-GFP), pRS315CUP-NM-M0-GFP (Sup35NM-M0-GFP), pRS315CUP-sup35-240-GFP (Sup35-240-GFP). Colocalization profiles were plotted using Image J. B. Estimation of colocalization rates. At least 50 cells from 3 replicates were counted. Mean and 95% confidence interval for the mean are shown. No significant difference in colocalization rates according to t-test could be observed.
