# Supplementary figures and images for: Processing of Fluorescent Proteins May Prevent Detection of Prion Particles in [PSI+] Cells
Source: Biology (Basel). 2022 Nov 22;11(12):1688. doi: 10.3390/biology11121688 (PMC9774836; doi:10.3390/biology11121688)

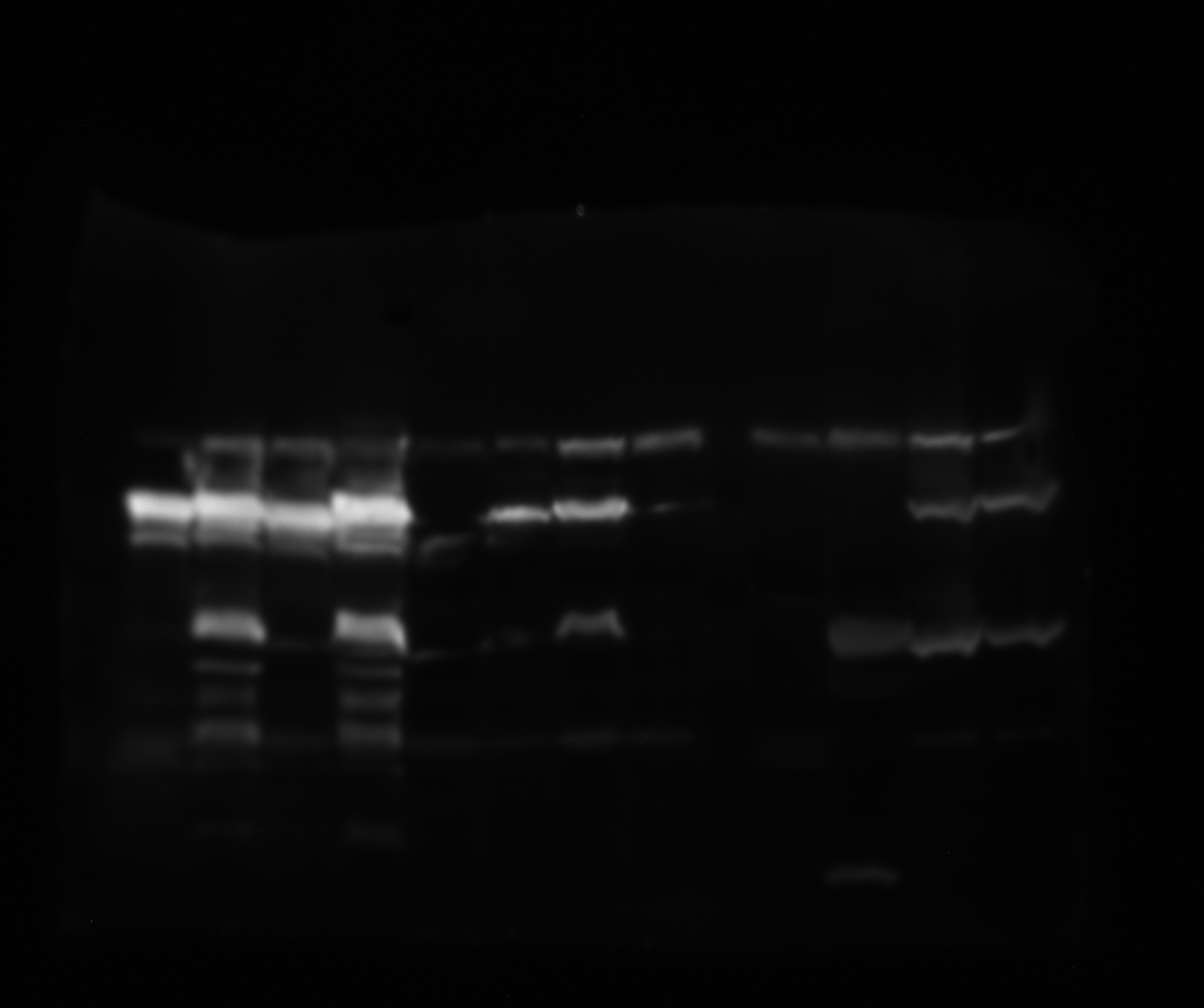

Supplement: Supplementary file 1 [file biology-11-01688-s001.zip › Fig. S3-Original Images of Fig3A.tif]

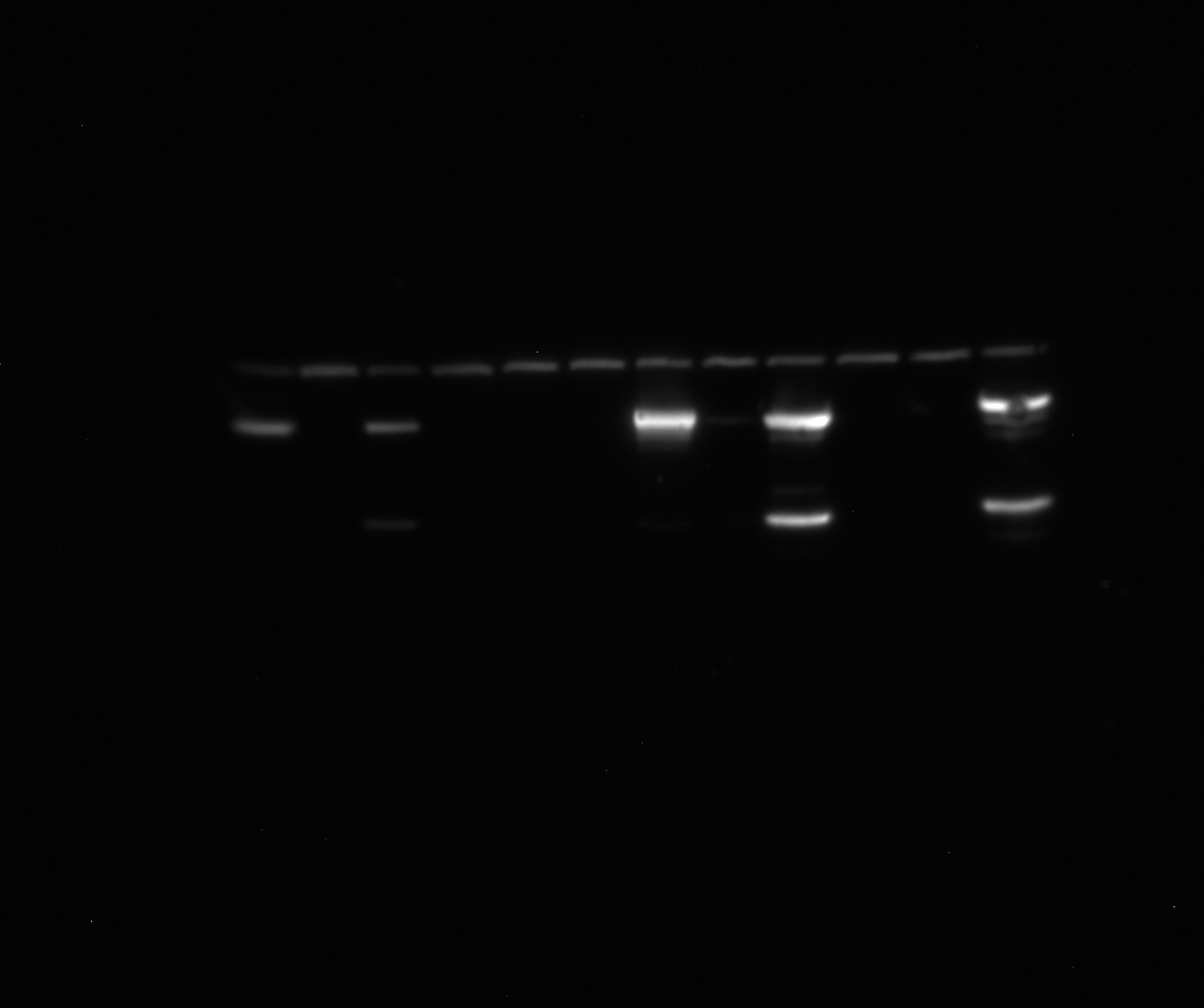

Supplement: Supplementary file 1 [file biology-11-01688-s001.zip › Fig. S4-Original Images of Fig3B(1).tif]

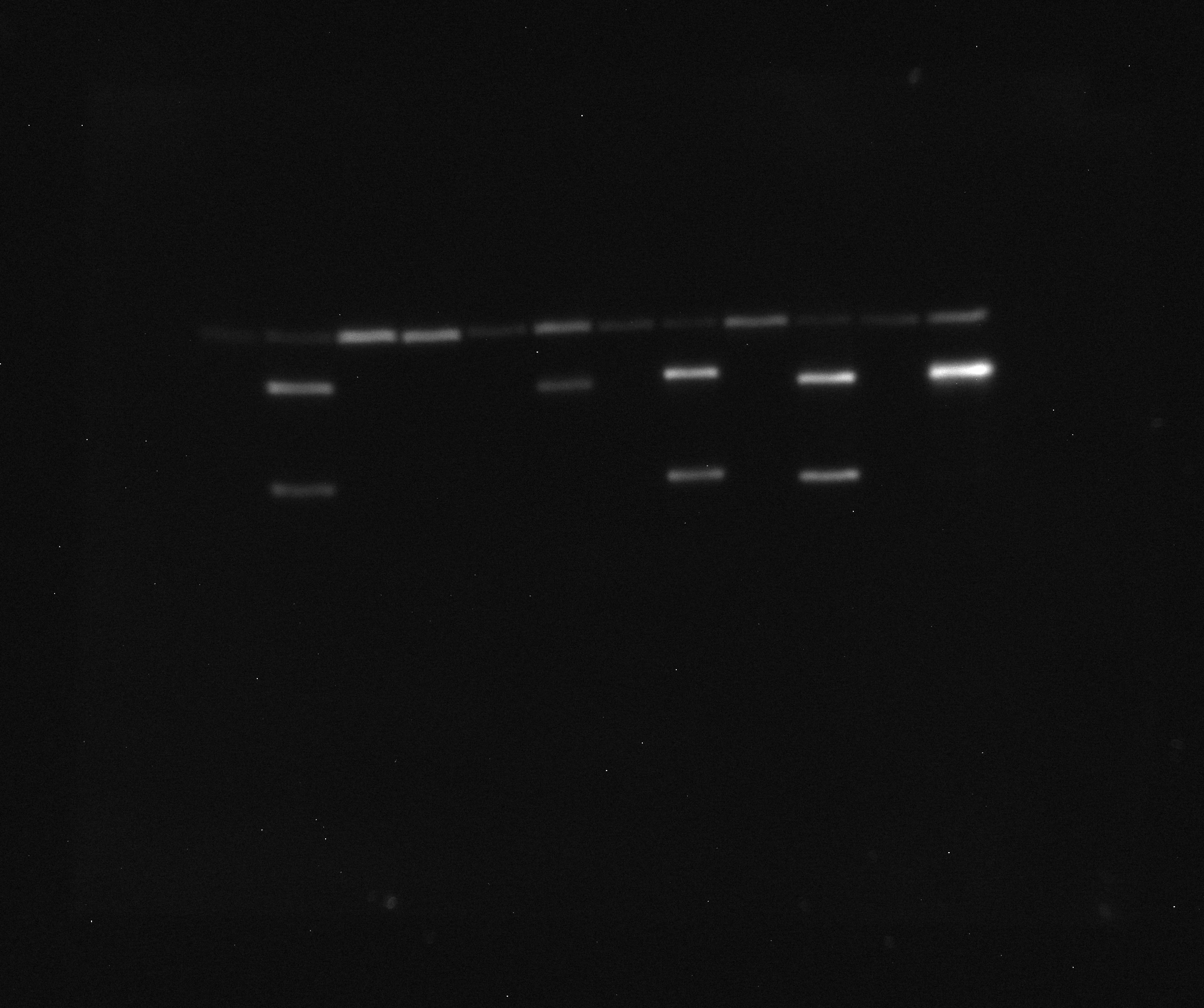

Supplement: Supplementary file 1 [file biology-11-01688-s001.zip › Fig. S5-Original Images of Fig3B(2).tif]

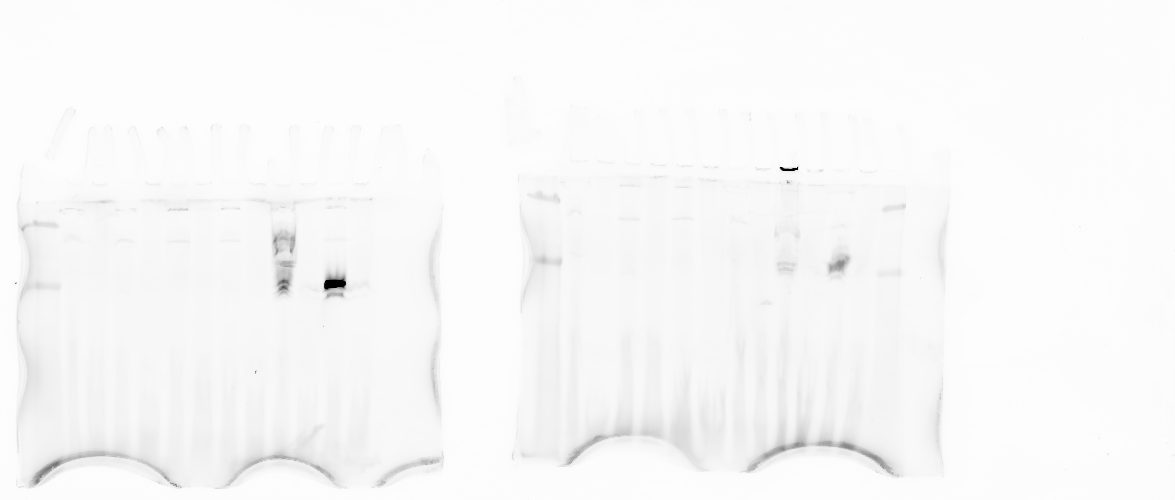

Supplement: Supplementary file 1 [file biology-11-01688-s001.zip › Fig. S6-Original Images of Fig4-[Alexa Fluor 488].tif]

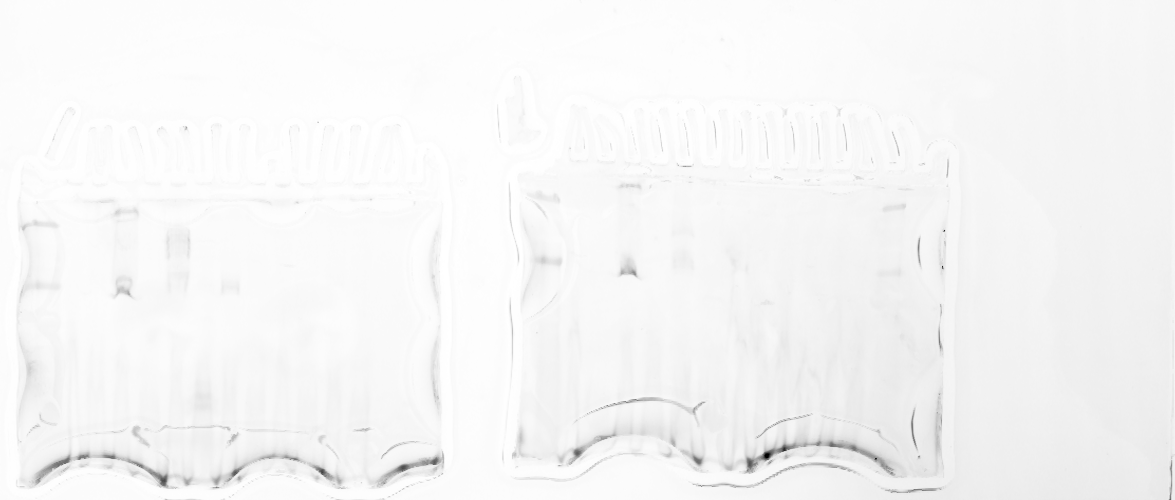

Supplement: Supplementary file 1 [file biology-11-01688-s001.zip › Fig. S7-Original Images of Fig4-[Cy3].tif]

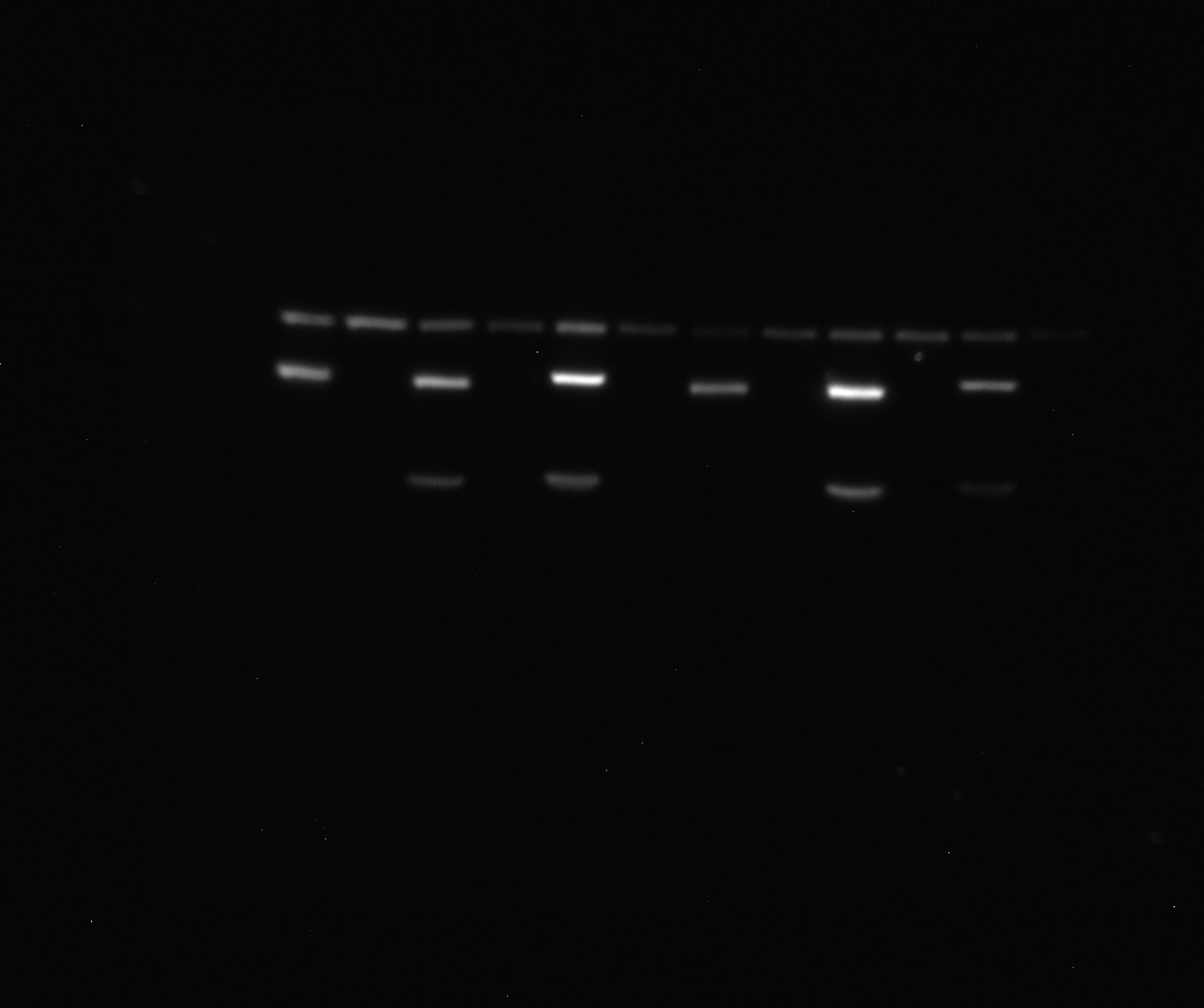

Supplement: Supplementary file 1 [file biology-11-01688-s001.zip › Fig. S8-Original Images of Fig5B.tif]

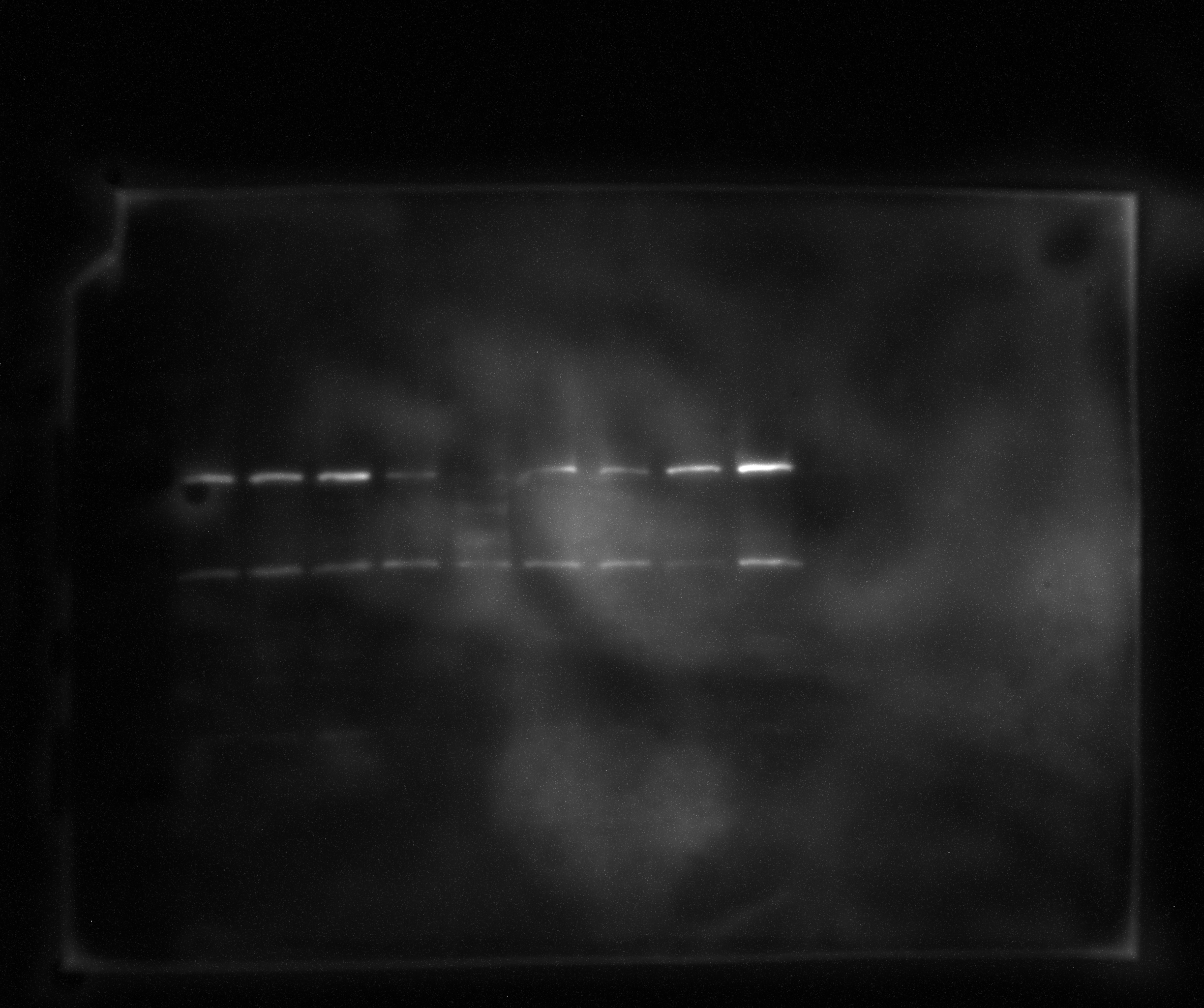

Supplement: Supplementary file 1 [file biology-11-01688-s001.zip › Fig. S9-Original Images of Fig6B.tif]
